# Deformation Control of Adjustable-Ring-Mode (ARM) Laser Welding for Aluminum Alloys

**DOI:** 10.3390/ma18040860

**Published:** 2025-02-16

**Authors:** Jinglong Tang, Minglie Hu, Jie Su, Qijun Guo, Xiaohua Wang, Zhen Luo

**Affiliations:** 1 Ultrafast Laser Laboratory, Key Laboratory of Opto-Electronic Information Technical Science of Ministry of Education, School of Precision Instruments and Opto-Electronics Engineering, Tianjin University, Tianjin 300072, China; 2 School of Materials Science and Engineering, Tianjin University, Tianjin 300350, China

**Keywords:** aluminum alloy, welding deformation, adjustable-ring-mode laser welding, beam quality

## Abstract

In the domain of new energy vehicles, the control of welding deformation in aluminum alloy battery systems poses substantial challenges. The existing methodologies for diminishing welding deformation, such as laser segmented skip welding, alteration of welding path sequences, numerical simulation prediction, and post-weld heat treatment, still possess room for further optimization when applied to intricate welding structures. In this research, a novel adjustable-ring-mode laser in conjunction with the oscillation welding technique was employed to explore the impacts of fiber core diameter, laser light field brightness distribution, and process parameters on weld formation. The regulation of welding deformation was achieved through optimizing the welding process and adjusting the welding path. The results indicate that when the fiber core diameter is 50/150 µm and the light field brightness distribution is H, the weld size exhibits the highest stability. Under the conditions of process parameters *p* = 5300 W, *v* = 5.4 m/min, *A* = 1.6 mm, *f* = 120 Hz, and *θ* = 40°, and with the spot position located at the bottom of the side of the upper substrate, the optimal weld formation is obtained. After optimizing the welding path, the maximum Z-direction deformation of the weld is 1.403 mm, representing a reduction of 1.702 mm compared to the previous value. This work is capable of providing novel theoretical guidance and technical insights for the control of welding deformation in thin aluminum alloy plates.

## 1. Introduction

Aluminum alloys are extensively utilized in aerospace, automotive manufacturing, and shipbuilding industries owing to their remarkable specific strength, favorable corrosion resistance, and low density [1,2]. Notably, in the context of new energy vehicles, aluminum alloys are prevalently utilized as the principal raw materials for battery trays [3,4,5]. This is attributed to their unique ability to facilitate weight reduction without compromising the structural strength, which holds paramount importance for battery electric vehicles. By enabling the downsizing of the battery system, aluminum alloys contribute significantly to cost reduction [6]. In the domain of vehicle body aesthetics, laser welding has emerged as the preferred joining technique. It is characterized by its capacity to produce high-quality surface finishes and visually appealing weld. Laser autogenous welding, in particular, has gained prominence as a lightweight joining solution in automotive applications by obviating the need for filler wire [7]. This technique offers distinct advantages in terms of processing efficiency and flexibility, which are primarily derived from the rapid positioning of welding spots and the non-contact single-sided energy input mechanism [8].

In recent years, the elevation of the pumping brightness of laser pump sources and the maturation of diverse fiber optic component technologies have led to the comprehensive improvement of the characteristics of fiber lasers. Lasing has been successfully achieved across a wide spectral range encompassing the ultraviolet, visible, near infrared, and mid-infrared regions [9]. By leveraging the master oscillator power amplifier (MOPA) technology [10] and high-quality, highly stable single longitudinal mode (SLM) fiber laser seed sources [11], it becomes feasible to fabricate an all-fiber high-power narrow-linewidth laser that approaches the diffraction limit. This laser exhibits a narrow linewidth, high output beam quality, and substantial power. Among the plethora of laser welding techniques, the adjustable-ring-mode (ARM) method has been recognized as an ideal approach for welding aluminum alloy battery trays. This is primarily attributed to its advantageous features, including high energy density, a high depth-to-width ratio, rapid welding speed, a narrow heat-affected zone, and minimal spatter [12,13].

The ARM laser is composed of an external annular beam (ring laser) and an internal Gaussian beam (core laser). The ring laser beam is capable of inducing a uniform temperature gradient on the material, thereby ensuring the stability of the welding process of the ring laser material and yielding a smooth surface of the material. Owing to the high intensity of the Gaussian laser beam, the core laser has been extensively utilized in deep penetration welding. Notably, the ARM laser effectively overcomes the drawbacks of the rough surface associated with Gaussian laser welding and the shallow penetration characteristic of annular laser welding [14]. Wang et al. [15] conducted experiments on stainless steel welding using the ARM laser. Their findings revealed that during high-speed welding, the ring laser served to stabilize the keyhole entrance, whereas the central laser generated a stable keyhole entrance as the welding speed increased. In the cases of dual ring/core and ring laser welding, the variation in the area of the keyhole entrance was primarily attributed to the alteration in length. In contrast, in core laser welding, the changes in area, length, and width were consistent. Sun et al. [7] investigated and verified the feasibility of employing the ARM laser technology to manipulate the microstructure of aluminum alloy welds. They determined that when combined with beam oscillation, the core/ring power ratio of the beam exhibited limited influence on the weld geometry. Specifically, increasing the oscillation width to 2.5 mm could enhance the joint strength; however, an excessively wide oscillation width would lead to a reduction in strength. An appropriate power ratio was found to effectively restrain the formation of columnar grains and facilitate grain refinement. Remarkably, when the power ratio was 0.33, the joint strength could attain 400 N/mm. In the realm of thin-sheet aluminum alloy welding, Xia et al. [16] conducted a comparative study between single-laser welding and ARM laser welding on ten-layer 0.4 mm-thick 5052 aluminum alloy sheets. Their findings indicated that the employment of ARM laser welding not only effectively mitigated welding deformation but also significantly enhanced the tensile strength of the welded joint. Concurrently, Venkat et al. [17,18] investigated the role of the ARM laser in aluminum alloy welding processes. Their research demonstrated that the ARM laser could refine columnar grains and promote the formation of secondary equiaxed grains, thereby effectively reducing the susceptibility to solidification cracking. When it comes to the welding of medium-thick aluminum alloy plates, studies [19,20,21] have shown that the ARM laser exhibits remarkable efficacy in reducing weld porosity. Specifically, an increase in the proportion of ring-beam power can enhance the stability of the keyhole, which in turn inhibits the generation of bubbles during the welding process. This contributes to a more stable welding process and improved weld quality. Furthermore, a multitude of studies [22,23,24] have initiated the application of the oscillation welding technique and optical coherence tomography detection technology in ARM laser welding. It has been demonstrated that in aluminum alloy welding, the ARM laser can significantly improve the weld surface morphology and augment the weld strength.

Nevertheless, there are relatively few reports regarding the deformation issue in the ARM laser welding process. In reality, in the welding of thin aluminum alloy plates, strong nonlinear phenomena frequently emerge [25]. During the welding procedure, the workpieces are partially melted and then promptly cooled down to room temperature. Throughout the welding thermal cycle, both the thermophysical and mechanical properties undergo significant variations with the change in temperature, which renders welding deformation highly susceptible. This, in turn, diminishes the dimensional precision of the fabricated structure and also gives rise to problems in the subsequent assembly process. Moreover, the welding sequence has a significant influence on welding deformation. Fahlström et al. [26] pointed out that the energy input of continuous laser welding is almost linearly related to the deformation. The research of Geng et al. [27] shows that the welding sequence plays a crucial role in the longitudinal and transverse residual stress distributions of U-shaped ribs. The continuous welding sequence leads to similar longitudinal and transverse residual stress distributions, while the segmented welding sequence shows characteristics of one high value and one low value. They also suggest using segmented double-sided welding to control the residual deformation. Finite element simulation analysis is also an effective way to predict welding deformation [28]. Huang et al. [29] have established linear and quadratic mathematical models between welding–warping deformation and welding parameters. These models can be used to predict the welding–warping deformation of laser-welded AA5052 aluminum alloy thin plates. The study finds that the larger the weld width, the greater the welding–warping deformation.

There exist several approaches to mitigate welding deformation, including laser segmented skip welding [30], altering the welding path sequence [31], numerical simulation prediction [25], and post-weld heat treatment [32].

Consequently, this study centers around thin aluminum alloy plate components. By integrating the ARM laser oscillation welding technique with laser segmented skip welding and optimized welding sequences, the emphasis is laid on the regulation of welding deformation. In this research endeavor, the influence of fiber core diameter and light field distribution on welding stability during the welding process is examined through an analysis of beam quality. Meanwhile, the effect of welding process parameters on weld formation is investigated by means of an assessment of weld morphology. This study is capable of furnishing both theoretical and practical references for the advancement of deformation control in ARM laser oscillation welding of aluminum alloys.

## 2. Materials and Methods

### 2.1. Materials

**Figure 1:**
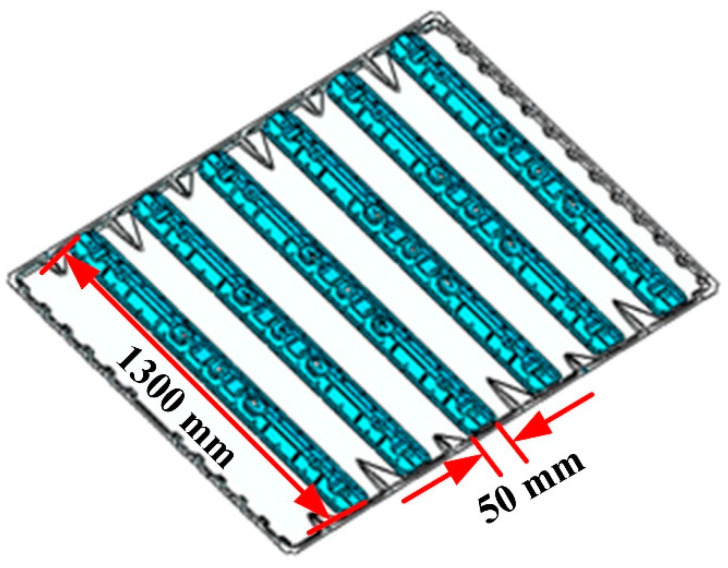
Schematic diagram of welding conditions.

In this research, the material utilized is 5182 aluminum alloy. The chemical composition and mechanical properties of this alloy are presented in Table 1 and Table 2, respectively. As illustrated in Figure 1, the working condition is composed of a single 1720 mm × 1330 mm × 3 mm bottom plate accompanied by six stiffeners.

Prior to the welding process, meticulous surface preparation of the aluminum alloy is carried out. Initially, the surface is cleaned with acetone and subsequently dried. This is followed by the removal of the surface oxide film using a grinding machine and sandpaper. Afterward, the specimens are rinsed with anhydrous ethanol. Finally, to eliminate the residual stress within the base plates and remove any moisture, the specimens are placed in a muffle furnace. They are heated to a temperature of 50 °C and maintained at this temperature for a duration of 2 h.

### 2.2. Experimental Procedure

**Figure 2:**
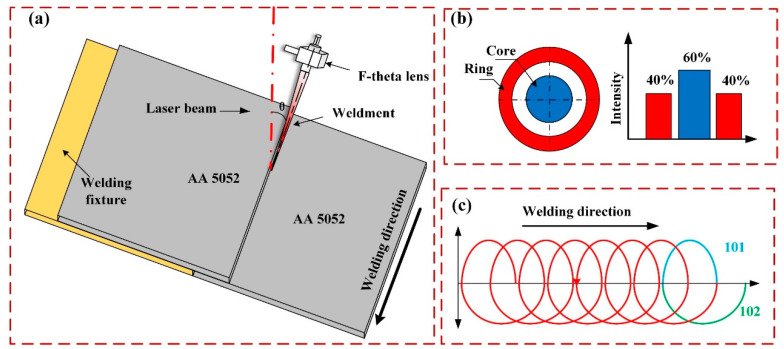
(**a**) Schematic diagram of ARM laser welding; (**b**) energy distribution of core/ring laser; (**c**) laser oscillation trajectory.

The ARM laser welding platform (Figure 2a) is composed of a fiber laser (YLS-AMB 6000/9000) manufactured by IPG Photonics Corporation (Marlborough, MA, USA), a D50 oscillating welding head, and two KUKA robots (KR60-2100, Augsburg, Bavaria, Germany). The fiber laser, serving as an adjustable-ring mode laser source, allows the core/ring fiber ratio to be set at either 50/150 µm or 100/300 µm (Figure 2b). Specifically, the maximum output power of the core beam reaches 9 kW, while that of the ring beam can attain 25 kW. The ARM laser encompasses the core and ring parts. To guarantee an optimal temperature distribution and the stability of the keyhole, the energy ratio between the core beam and the ring beam is typically maintained at 6:4, as reported in reference [22]. During the welding process, a counterclockwise circular oscillating beam trajectory is adopted. The motion path and instantaneous velocity of the beam are defined by Equations (1) and (2), respectively.

(1) $$\left\{\begin{array}{l} x(t)=x_{0}+\frac{A}{2}\times \cos(2\pi \times f\times t)+v\times t\\ y(t)=y_{0}+\frac{A}{2}\times \sin(2\pi \times f\times t) \end{array}\right.$$

(2) $$\left\{\begin{array}{l} V_{x}(t)=x^{\prime }(t)=-A\times \pi \times f\times \sin(2\pi \times f\times t)+v\\ V_{y}(t)=y^{\prime }(t)=A\times \pi \times f\times \cos(2\pi \times f\times t)\\ V=\sqrt{V_{x}^{2}+}V_{y}^{2} \end{array}\right.$$

Here, *x*_0_ and *y*_0_ are the initial positions of the laser spot in space. *f* and *A* represent the oscillation frequency and the oscillation amplitude, respectively. *t* is the welding duration, and *v* represents the instantaneous welding speed. The initial position of the laser focus is precisely set on the base material. Within the unit waveform of the beam’s movement path, the first arc trajectory segment, 101, characterized by a relatively small radius of curvature, exerts its influence on the upper plate. Conversely, the second arc trajectory segment, 102, featuring a relatively large radius of curvature, acts upon the lower plate. The scanning strategy is presented as depicted in Figure 2c.

Initially, the lap joint configuration is adopted to preliminarily explore the welding process. The welding process is depicted in Figure 2. Throughout the welding process, the defocusing amount was precisely maintained at 0 mm. The shielding gas employed was 99.999% pure argon (Ar) with a flow rate of 25 L/min. To conduct a comprehensive and in-depth investigation into the influence of various process parameters, a single-variable testing approach was systematically adopted. Table 3 meticulously lists the parameters of laser power, welding speed, amplitude, frequency, beam tilt angle, and spot position that were utilized in this research endeavor.

### 2.3. Analysis Methods

To analyze the influence of factors such as the laser light and optical path collimation on the generated ARM light, and to avoid external factors from affecting the subsequent welding quality, a beam quality measuring instrument (Primes Focus Monitor, Pfungstadt, Hesse, Germany) was used to measure the beam quality of the light beam generated by this ARM light welding system. A bridge-type coordinate measuring machine (ZEISS, CONTRA, Carl Zeiss AG, Oberkochen, Baden-Württemberg, Germany) was adopted to precisely measure the deformation of the characteristic regions subsequent to welding. Measuring accuracy is specified as (2.2 + 3*l*) mm, with *l* denoting the length of the measured specimen. For each set of data, three repeated measurements are carried out, and the average value is calculated.

After welding, the specimens undergo computer numerical control (CNC) machining. Subsequently, metallographic specimens are meticulously prepared by employing Keller’s reagent, which consists of 1% HF, 1.5% HCl, 2.5% HNO₃, and 95% H₂O as the etchant. The microstructure of the laser-welded joints of the Al alloy is then examined using a metallographic microscope (DM4000M, manufactured by Leica, Wetzlar, Germany).

## 3. Results and Discussion

### 3.1. Effect of Optical Fiber Core Diameter

**Figure 3:**
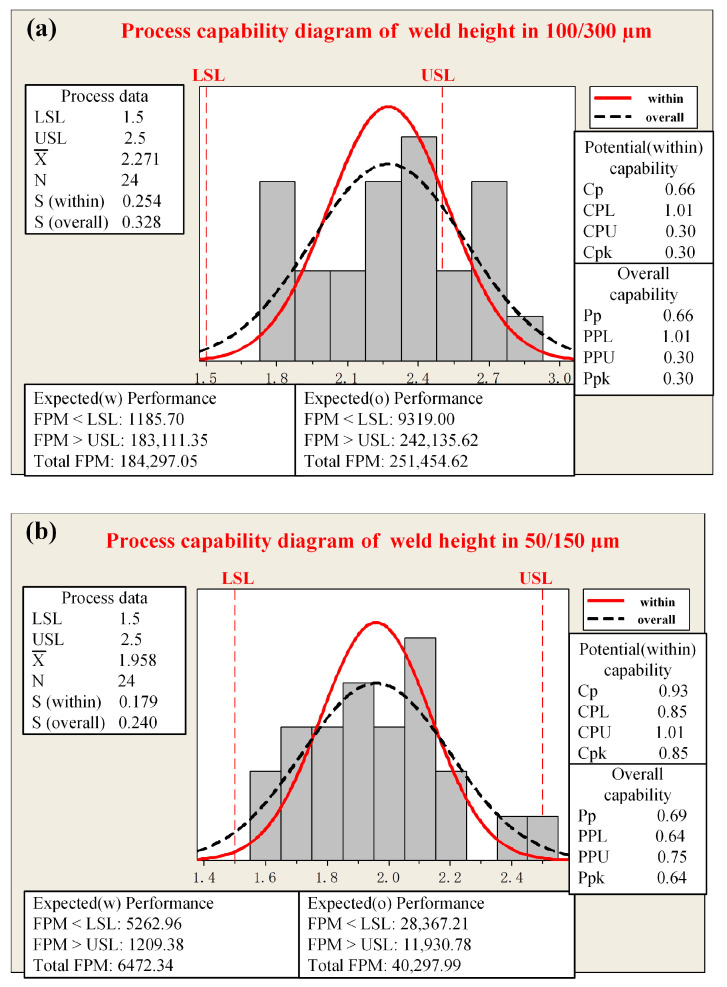
Process capability diagram of weld heights with different fiber core diameters: (**a**) 100/300 µm; (**b**) 50/150 µm.

**Figure 4:**
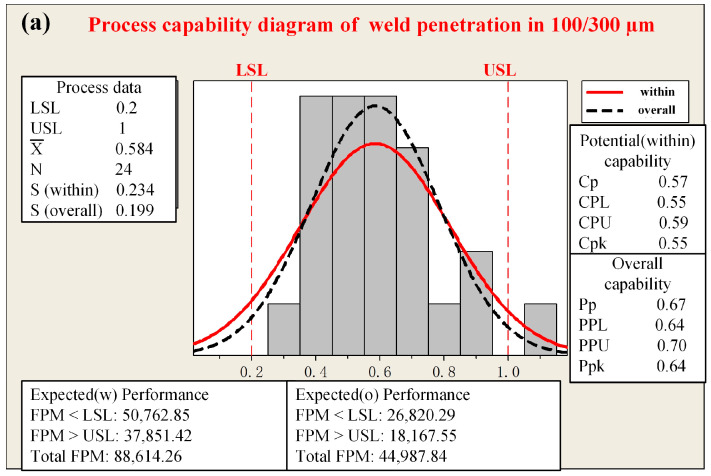

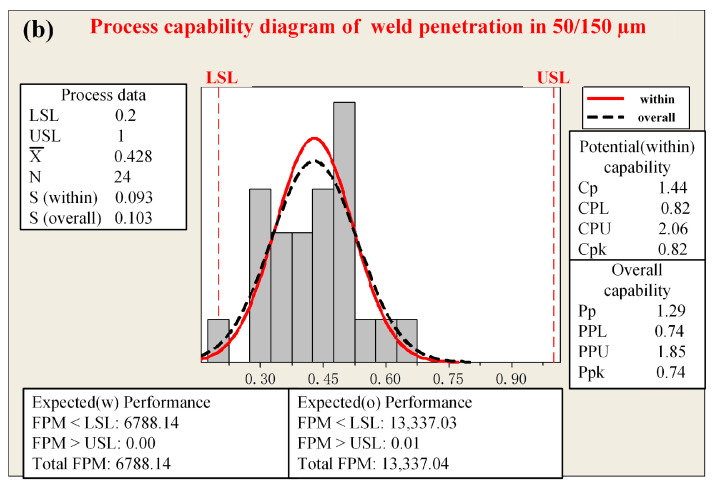
Process capability diagram of weld penetration with different fiber core diameters: (**a**) 100/300 µm; (**b**) 50/150 µm.

Systematic measurements of post-welding height and penetration depth were carried out for fiber core diameters of 100/300 µm and 50/150 µm, where 100 and 50 represent the core diameters of the central fibers, and 300 and 150 denote the core diameters of the ring fibers. The analysis and plotting were carried out using Minitab software (Minitab 20), and the specific results are shown in Figure 3 and Figure 4. For each of these two distinct fiber core diameter configurations, a series of 24 tests were meticulously performed. The computational methodologies for the relevant parameters, as referenced in [33], are explicitly illustrated in Equations (3)–(5).

(3) $$\left\{\begin{array}{l} Cp=\frac{USL-LSL}{6S_{within}}\\ CPL=\frac{\bar{X}-LSL}{3S_{within}}\\ CPU=\frac{USL-\bar{X}}{3S_{within}}\\ Cpk=\min\left(CPL,CPU\right) \end{array}\right.$$

(4) $$\left\{\begin{array}{l} Pp=\frac{USL-LSL}{6S_{overall}}\\ PPL=\frac{\bar{X}-LSL}{3S_{overall}}\\ PPU=\frac{USL-\bar{X}}{3S_{overall}}\\ Ppk=\min\left(PPL,PPU\right) \end{array}\right.$$

(5) $$\left\{\begin{array}{l} FPM_{within}=\left(1-\frac{N_{fail,within}}{N_{within}}\right)\times 100\%\\ FPM_{total}=\left(1-\frac{N_{fail,total}}{N_{total}}\right)\times 100\% \end{array}\right.$$

where *Cp* represents the process capability ratio, *CPL* denotes the lower capability index, and *CPU* is the upper capability index. *Cpk* represents the process capability index with offset, which is an indicator used to measure the actual process ability. A larger *Cpk* indicates that the process can satisfy both the lower and upper specification limits, suggesting a stronger overall process capacity. *USL* and *LSL* are the upper and lower specification limits, respectively, with *S* representing the standard deviation. $\bar{X}$ represents the sample mean, *S_within_* usually refers to the within-group standard deviation, *S_overall_* generally refers to the overall standard deviation. *Pp* represents the process potential capability, assuming the process means are centered within the specification limits. *PPL* is the lower-bound process potential capability, relating to the process mean and the lower specification limit. *PPU* is the upper-bound process potential capability, concerning the process mean and the upper specification limit. *Ppk*, the actual process capability index, measures the real-life process capability. Notably, a higher *Ppk* indicates better process capability and more stable product quality, with a reduced likelihood of non-conformities. The within-group capability is computed via Equation (3), and the overall capability through Equation (4). stands for the first-pass yield in production, and *N* represents the sample size. *N_fail,within_* refers to the number of non-conforming products within the subgroup, and *N_within_* is the total number of products within the subgroup. *FPM_within_* calculates the percentage of conforming products within the subgroup samples, which is used to evaluate the quality level of the production process within the subgroup. *N_fail,total_* represents the total number of non-conforming products, and *N_total_* is the total number of all products. *FPM_total_* calculates the percentage of conforming products among all products, reflecting the overall quality status of the entire production process.

The mean weld height measures 2.27 mm when the fiber core diameter of 100/300 µm is employed, whereas it amounts to 1.96 mm when utilizing the fiber core diameter of 50/150 µm. Evidently, the mean weld height of the latter configuration exceeds that of the former. In terms of the weld height, both the *Cpk* value (0.30) and the *Ppk* value (0.85) associated with the 100/300 µm fiber core diameter is lower than those corresponding to the 50/150 µm fiber core diameter. This disparity clearly indicates that the weld height achieved with the 50/150 µm fiber core diameter is more stable. The outcomes of the weld penetration are presented in Figure 4. When the fiber core diameter of 100/300 µm is utilized, the mean weld penetration is 0.58 mm, while it is 0.43 mm for the 50/150 µm fiber core diameter. Nevertheless, upon comparing the *Cpk* and *Ppk* values of the weld penetration, it is discerned that the fiber core diameter of 50/150 µm demonstrates a more stable performance. Considering the combined results of both the weld height and penetration, the fiber core diameter of 50/150 µm is deemed the optimal choice for subsequent weld deformation control studies.

**Figure 5:**
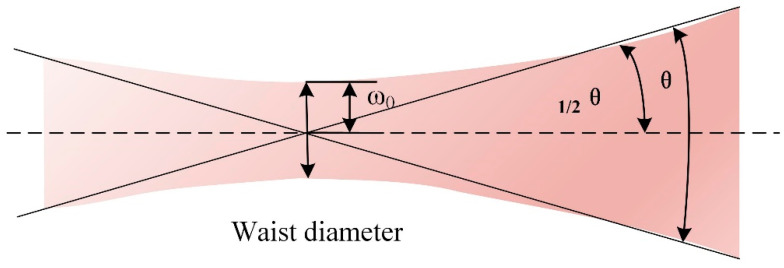
Diagram of beam geometry parameters.

In the realm of fiber lasers, the beam parameter product (BPP) serves as a pivotal parameter for quantifying the quality of the laser beam, exerting a direct influence on the quality of the laser welding processes. The BPP of a laser is constant. When the beam waist or focal point is augmented via an optical system, the divergence angle correspondingly diminishes. Conversely, an increase in the divergence angle leads to a reduction in the size of the beam waist or focal point, as depicted in Figure 5. The BPP can be straightforwardly calculated using the beam waist size and the far-field divergence angle. This value provides insights into the beam’s propagation characteristics and focus ability. As detailed in reference [34], the specific calculation is presented in Equations (6)–(8).

(6) $$BPP=\omega_{0}\times \theta$$

(7) $$\omega_{0}=\frac{D}{2}=R$$

(8) θ=sinα=α=NA

Here, *ω*_0_ represents the beam waist radius, and *θ* denotes the far-field divergence angle of the beam. *D* is the core diameter of the optical fiber, and *NA* is the numerical aperture of the optical fiber.

**Figure 6:**
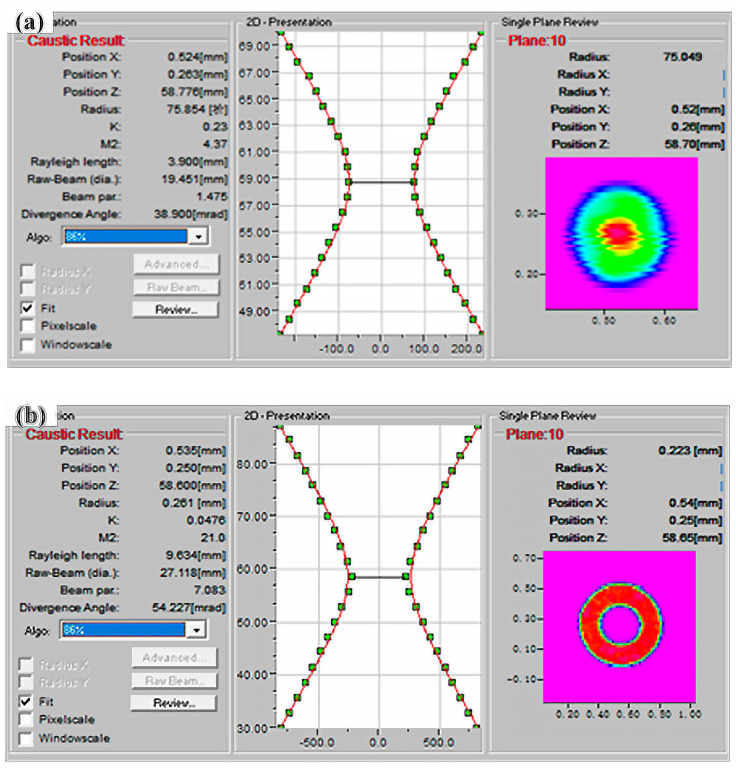
Results of the beam quality: (**a**) core laser; (**b**) ring laser.

An experimental investigation was conducted to assess the beam quality of a laser featuring an optical fiber core diameter of 50/150 µm. The beam quality of the beam passing through the ARM welding optical system was measured and recorded at the focal plane and in the vicinity of the focal plane, respectively. For the focused light with a focal length of 210 mm, the beam quality within the range of ±5 mm from the focus on the optical axis was obtained, and the results are shown in Figure 6. Regarding the core laser, the M^2^ factor was determined to be 4.37, the *θ* measured 38.9°, and the BPP amounted to 1.475. In contrast, for the ring laser, the M^2^ value reached 21.0, the *θ* was 54.227°, and the BPP was calculated as 7.083. The core laser exhibits superior beam quality, attributable to the relatively small diameter of the optical fiber. The energy distribution of the light spots for both lasers follows a pattern where the energy is higher at the center and lower at the peripheries, approximating a Gaussian distribution. Specifically, at the focal plane, the spot radius attains its minimum value, while the peak power reaches its maximum. As the defocus distance increases, there is a corresponding increase in the spot radius, accompanied by a decrease in the peak power of the spot.

### 3.2. Effect of Light-Field Distribution

The light-field distribution primarily pertains to the distribution patterns of the light field within a laser. These patterns are stable forms of light-field distribution that are generated in the optical resonator of the laser. In the three directions of the laser, namely, perpendicular to the PN junction, parallel to the PN junction, and along the optical axis, the light field must fulfill the resonance conditions to form a standing-wave distribution. During the transmission process, the laser beam may be subject to effects such as scattering, absorption, and interference, which can lead to alterations in the light-field distribution.

**Figure 7:**
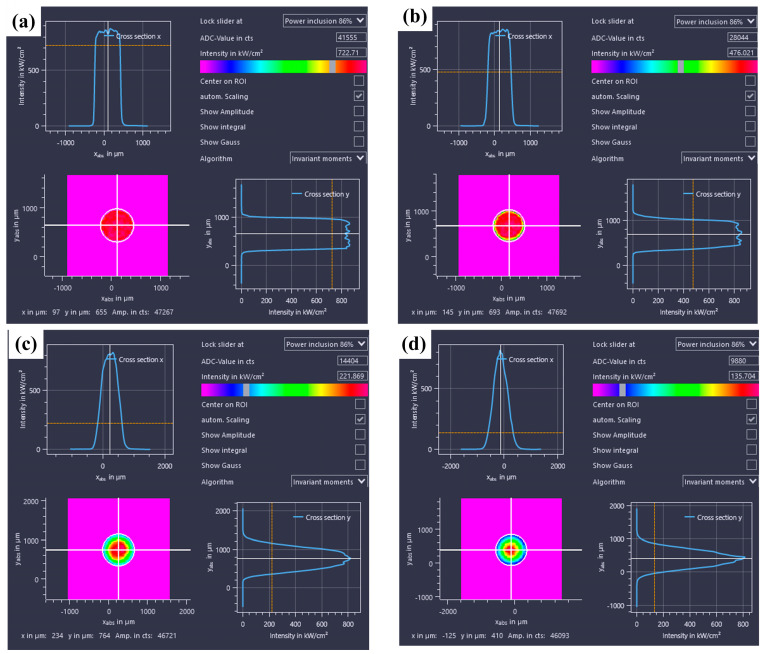
Energy density diagram of different light-field distributions: (**a**) H; (**b**) HH; (**c**) HHH; (**d**) HHHH.

In this research, an in-depth investigation is conducted on four distinct light-field distributions (Table 4). The energy density distributions were measured using a laser focus analyzer (Focus Monitor FM+), as depicted in Figure 7. Specifically, the energy distributions along both the *X*-axis and the *Y*-axis demonstrate a characteristic pattern where the values are higher at the center and gradually decrease towards the two sides, conforming to an overall Gaussian distribution. Notably, as the light-field configuration evolves from H to HHHH, there is a concomitant increase in the degree of concentration of the laser energy distribution. Nevertheless, in practical welding applications, it has been found that the degree of laser energy distribution does not exhibit a straightforward positive correlation with the welding performance.

**Figure 8:**
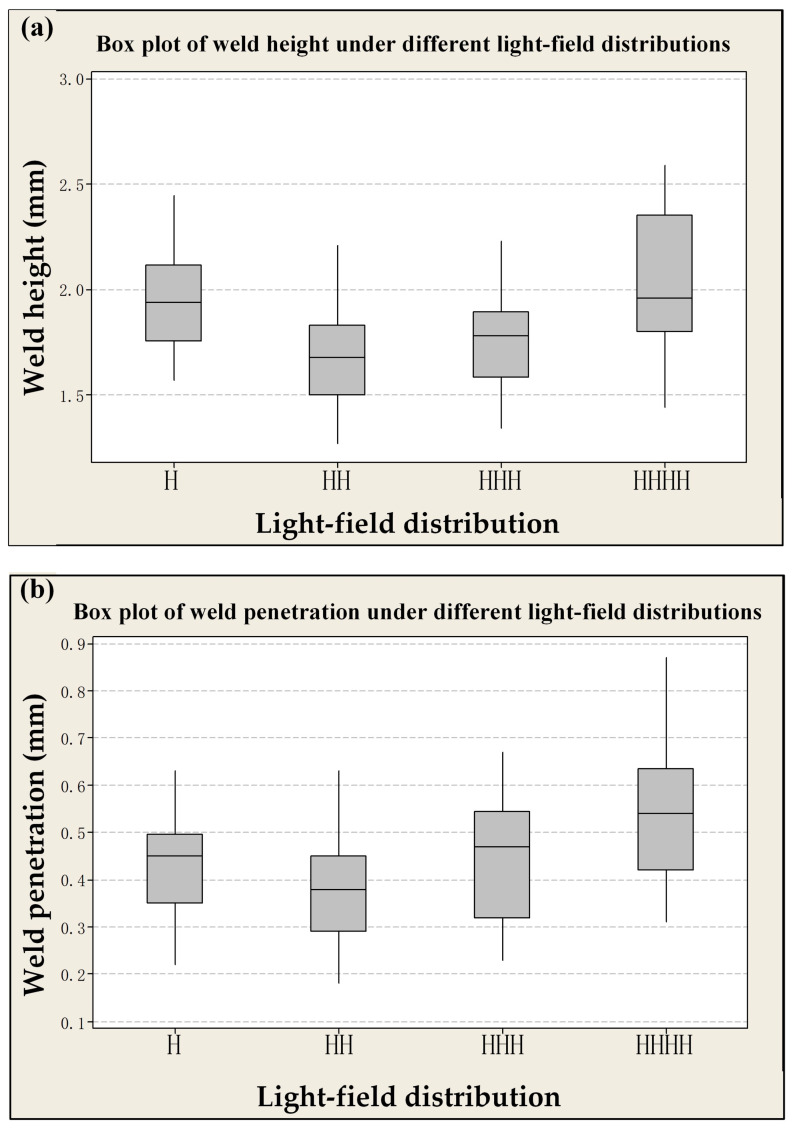
Box plot of different light-field distributions: (**a**) weld height; (**b**) weld penetration.

Figure 8 depicts, in the format of box plots, the impact of four distinct light-field distributions on laser weld formation under identical process parameters. Concerning the weld height, the H and HHHH light-field distributions yield relatively high weld heights, measuring approximately 1.95 mm. The weld heights for the HH and HHH light-field distributions are approximately 1.6 mm and 1.7 mm, respectively. Notably, for the weld heights associated with HH, HHH, and HHHH, the lower whiskers are less than 1.5 mm. This indicates the presence of non-compliant data within the weld dimensions. The interquartile range of the weld height for HHHH is the largest among them, signifying that the degree of variability in the weld height of this particular laser is the greatest, with pronounced fluctuations. In contrast, the weld height dimensions of H are in full compliance with the requirements. Regarding the weld penetration, the weld penetration of HHHH is the most substantial, surpassing 0.5 mm. However, the interquartile ranges of the weld penetration for HHH and HHHH are relatively large. This implies that the degree of variance in the weld penetration of these lasers is significant, resulting in notable fluctuations. Moreover, the lower whisker of the weld penetration for HH is less than 0.2 mm, suggesting the existence of non-compliant data in the weld dimensions. Conversely, the weld penetration dimensions of H satisfy the requirements, demonstrating relatively good stability in the weld dimensions. Despite the fact that the weld penetration and height increase in tandem with the rise in laser energy density, the fluctuations in the weld dimensions are considerable, leading to an unstable welding effect. To sum up, both the weld height and the penetration of H meet the specified requirements, and the welding stability is at an optimal level. Consequently, this light-field distribution pattern will be employed in subsequent experiments.

### 3.3. Effect of Welding Technology Parameters

Firstly, a comparative test of two welding processes, specifically, low-power and slow-speed welding (with *p* = 2650 W and *v* = 2.7 m/min) and high-power and fast-speed welding (with *p* = 5300 W and *v* = 5.4 m/min), is carried out under identical conditions (where *A* = 1.6 mm, *f* = 120 Hz, *θ* = 40°, and the laser spot is positioned at the middle of the side of the upper substrate). Based on Equation (9) [35], the welding heat input for both processes is calculated to be 59.6 J/mm.

(9) $$Q=\frac{\eta p}{v}$$

where *Q* is the heat input per unit length of weld in J/mm, the values of η range from 0.80 to 0.95, *p* is the laser power in W, and *v* is the welding speed.

**Figure 9:**
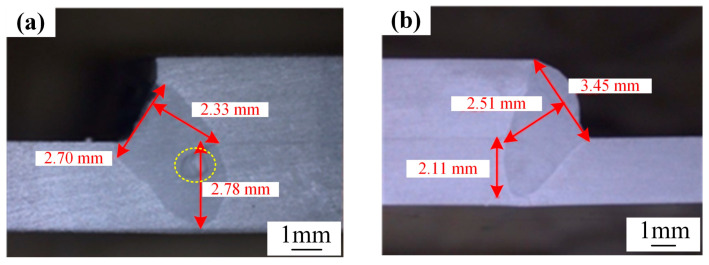
Weld cross-sectional morphology: (**a**) low-power and slow-speed welding; (**b**) high-power and fast-speed welding.

The cross-sectional morphology of the weld is presented in Figure 9. For the low-power and slow-speed welding process, significant pores are observed within the weld. Conversely, in the case of the high-power and fast-speed welding process, the weld exhibits a favorable formation. However, its penetration is marginally lower than that of the low-power and slow-speed welding process.

**Figure 10:**
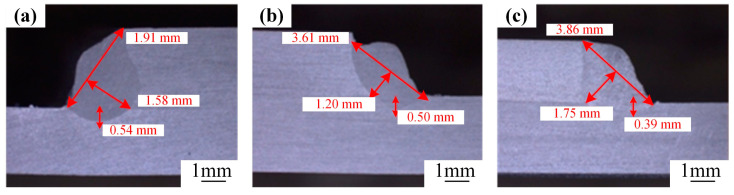
Weld cross-sectional morphology with different oscillation frequencies: (**a**) 200 Hz; (**b**) 250 Hz; (**c**) 300 Hz.

The high-power and fast-speed welding process is chosen to explore the influence of the oscillation frequency. During this research, the welding parameters are kept constant: *p* = 5300 W, *v* = 5.4 m/min, *A* = 2.0 mm, and *θ* = 20°, and the laser spot is located precisely in the middle of the side of the upper substrate. Figure 10 depicts the cross-sectional morphologies of the welds corresponding to different frequencies. Through careful observation and analysis, it has been determined that an increment in the frequency leads to a further reduction in the weld penetration. Nevertheless, it exerts a positive impact on broadening the weld width.

**Figure 11:**
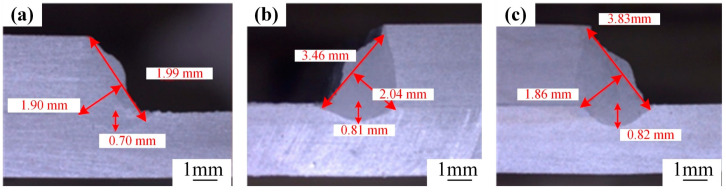
Weld cross-sectional morphology with different spot positions: upper (**a**), middle (**b**), lower (**c**) parts of the upper plate’s side.

An in-depth investigation into the laser spot position is carried out. The laser spot is positioned at the upper, middle, and lower parts of the side of the upper substrate for a comparative analysis. Figure 11 illustrates the outcomes of the cross-sectional morphologies of the welds corresponding to these three positions (*p* = 5300 W, *v* = 5.4 m/min, *A* = 2.0 mm, *f* = 200 Hz, *θ* = 20°). Through meticulous observation and analysis, it has been revealed that when the laser spot impinges on the lower side of the upper substrate, it facilitates an increase in the weld penetration. Nevertheless, the weld morphologies under these three positions exhibit no substantial variations.

**Figure 12:**
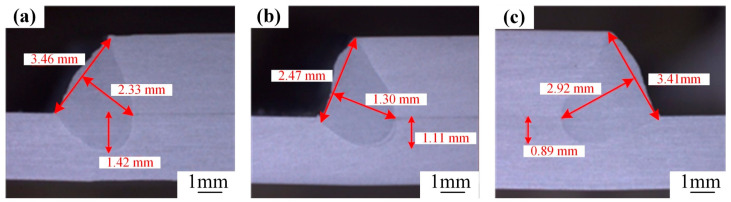
Weld cross-sectional morphology with different tilt angles: (**a**) *θ* = 20°, (**b**) *θ* = 30°, (**c**) *θ* = 40°.

Nonetheless, the weld penetration under the aforementioned processes are all remarkably shallow. Hence, with the laser spot located at the bottom of the side of the upper substrate, an in-depth exploration is carried out by fine-tuning the angle of the laser welding torch. Figure 12 depicts the cross-sectional morphologies of the welds corresponding to various welding torch inclination angles (*p* = 5300 W, *v* = 5.4 m/min, *A* = 1.6 mm, *f* = 120 Hz). Through comprehensive analysis, it is evident that upon optimizing the process parameters, a substantial enhancement in the weld penetration is achieved. Specifically, as the inclination angle of the welding torch gradually increases, the weld penetration experiences a corresponding increase. The maximum weld penetration depth is attained when *θ* = 40°.

**Figure 13:**
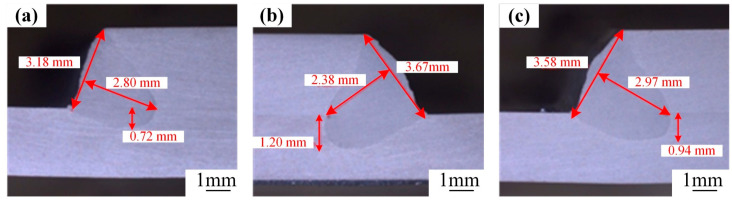
Weld cross-sectional morphology with different oscillation amplitudes: (**a**) 1.2 mm, (**b**) 1.6 mm, (**c**) 2.0 mm.

The impact of the oscillation amplitude on weld formation is depicted in Figure 13. With the progressive increase in the oscillation amplitude, the weld penetration depth initially exhibits an upward trend and subsequently a downward one. The peak value of the weld penetration is attained when *A* = 1.6 mm. To sum up, under the parameters of *p* = 5300 W, *v* = 5.4 m/min, *A* = 1.6 mm, *f* = 120 Hz, and *θ* = 40°, when the laser spot is located at the lower side of the upper substrate, a lap joint is achieved, featuring no discernible defects and demonstrating excellent weld formation.

### 3.4. Effect of Welding Path

**Figure 14:**
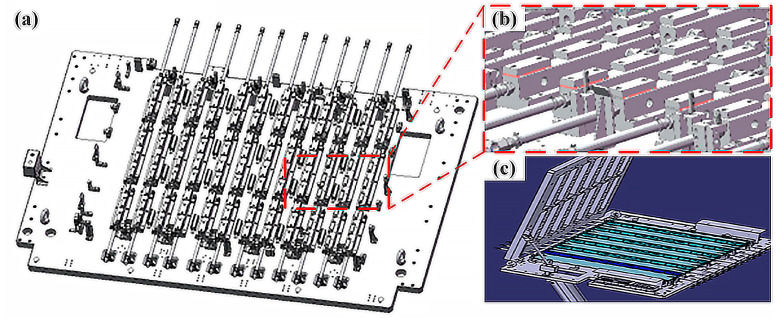
(**a**) Overall design of the welding fixture; (**b**) partial enlargement of the fixture; (**c**) Schematic diagram of the anti-deformation plate.

Upon the determination of the process parameters, and subsequent to the design of the fixture and anti-deformation plate, as depicted in Figure 14, the welding operation is carried out on the workpiece presented in Figure 1. The fixture is equipped with both water-cooling and air-cooling modules. The water-cooling apparatus offers circulating cooling throughout the welding process. As for the air-cooling device, it serves a dual purpose. On one hand, it can be utilized to lower the temperature of the weld; on the other hand, it is capable of removing the welding slag adhering to the surface of the aluminum alloy.

**Figure 15:**
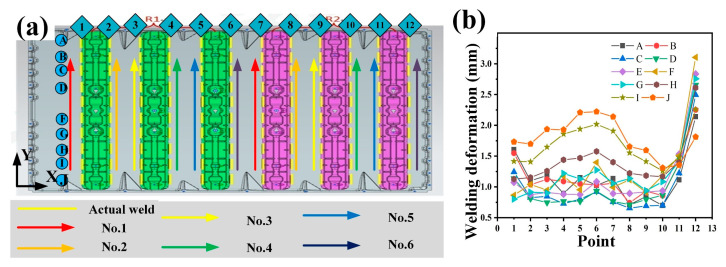
(**a**) Welding schematic diagram of Path 1; (**b**) welding deformation curve of Path 1.

Three distinct welding paths are utilized to accomplish the welding procedure. Subsequently, a comprehensive comparison is conducted to assess the impacts of these welding paths on welding deformation. Figure 15 depicts the welding approach of Path 1 and showcases the meticulously collected welding deformation data at various positions. In Figure 15a, arrows of diverse colors denote the welding sequence. Specifically, the red arrow symbolizes the first weld, and the yellow arrow represents the second weld. During the welding operation, two robots are deployed to work concurrently. Robot 1 undertakes the welding task of the green-colored area, while Robot 2 is assigned to weld the pink-colored area. The yellow straight line in the figure designates the actual location of the weld. To mitigate deformation, an intermittent welding technique is implemented. Figure 15b presents the collected welding deformation measurements at 12 positions (points 1–12) along the *X*-axis and 10 positions (A–J) along the *Y*-axis. The results reveal that the welding deformation associated with Path 1 is predominantly concentrated at the No. 6 weld (point 6 and point 12). The maximum deformation attains a value of 3.105 mm, and the overall deformation exhibits a pronounced tendency to propagate in the X-direction. Through in-depth analysis, it is postulated that the heat generated during the welding process migrates towards the X-direction, where the temperature is relatively lower. This leads to an asymmetrical heat input on either side of the base plate, ultimately resulting in the occurrence of welding deformation in the base plate. Intriguingly, the deformation manifested under Path 1 is a positive deformation, characterized by an upward bulge.

**Figure 16:**
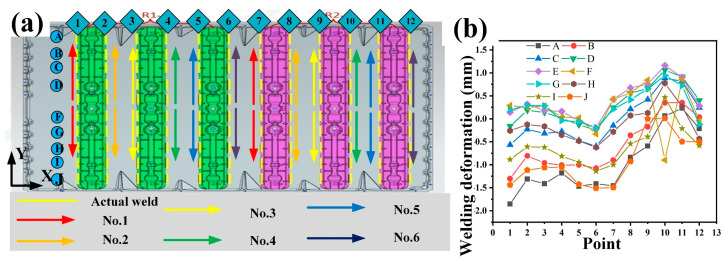
(**a**) Welding schematic diagram of Path 2; (**b**) welding deformation curve of Path 2.

The welding methodology and associated welding deformation characteristics of Path 2 are illustrated in Figure 16. In contrast to Path 1, the initiation point of the welding process for Path 2 is relocated from the bottom of the *Y*-axis to the mid-point of the *Y*-axis. Subsequently, the welding operation proceeds bilaterally along the *Y*-axis. As depicted in Figure 16b, the welding deformation curve exhibits a wavy pattern along the *X*-axis. This curve is characterized by a depression on one side and a bulge on the other. The maximum value of the welding deformation, which is −1.853 mm, is observed in the upper-left corner of the workpiece. Nevertheless, when compared to Path 1, a substantial improvement in the welding deformation of Path 2 is evident.

**Figure 17:**
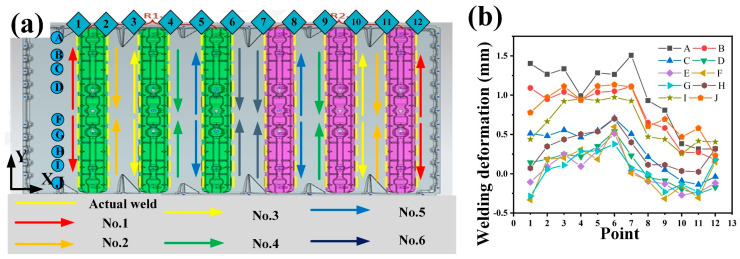
(**a**) Welding schematic diagram of Path 3; (**b**) welding deformation curve of Path 3.

The welding approach of Path 3, as depicted in Figure 17a, builds upon the foundation of Path 2, with a strategic modification to the welding sequence of the two robots. Specifically, the welding process commences with operations from both lateral sides of the workpiece. This bilateral welding is meticulously executed to ensure an even distribution of heat input at the initial stage, thereby minimizing potential thermal stress concentration. Subsequently, the final stage of the welding operation focuses on the central part of the workpiece. This sequential approach not only considers the heat-related effects during welding but also optimizes the overall integrity of the welded structure. Moreover, the welding path has been reconfigured into a “circular” pattern, which implies a more continuous and systematic welding process, facilitating better control over the heat dissipation and material flow. The outcomes of the welding deformation under Path 3 are presented in Figure 17b. Through comprehensive analysis of the data, it is evident that the overall deformation is confined within a relatively narrow range, specifically from −0.285 mm to 1.403 mm. This indicates that the implementation of this welding path has led to a substantial reduction in welding deformation. In fact, it has essentially achieved an ideal state of deformation regulation, demonstrating its effectiveness in enhancing the quality and dimensional stability of the welded components.

## 4. Conclusions

In the present research, a novel ARM laser was employed to conduct welding operations on aluminum alloy specimens. The regulation of welding deformation was accomplished through a series of strategies. These included the meticulous selection of the fiber core diameter and the precise manipulation of the laser light field brightness distribution. Additionally, the welding process parameters were optimized, and the welding path was adjusted with great care. The specific conclusions are as follows:

(1) Using the process capability diagram to evaluate the optical fiber core diameter’s impact, it was found that a 50/150 µm core diameter offers more stable welding. The Cpk and Ppk values for weld penetration and height are higher. The BPP values are 1.475 (core laser) and 21.0 (ring laser). When the light field luminance distribution is H, weld dimensional stability is maximized, ensuring optimal welding.
(2) The effects of oscillation frequency, amplitude, tilt angles, and spot position on weld dimensions were studied. It was determined that at *p* = 5300 W, *v* = 5.4 m/min, *A* = 1.6 mm, *f* = 120 Hz, and *θ* = 40°, with the spot at the bottom of the upper substrate’s side, a lap joint with few defects and good weld formation can be obtained.
(3) The influence of different welding paths on weld deformation was examined. After optimization, the maximum *Z*-axis weld deformation decreased to 1.403 mm, a reduction of 1.702 mm. During cross-symmetric welding, the second weld’s deformation offsets part of the first weld. The closer the symmetric welds are to the outer part, the smaller the post-welding deformation peak, enabling effective control of overall post-welding deformation.
(4) In future research, this work can be combined with real-time monitoring and feedback control of the welding process. Further research on the compatibility of ARM laser welding with newly developed thin aluminum alloys should be carried out to ensure that the technology remains applicable and effective in the face of the ever-changing material demands in the industry. In this way, its strong industrial potential, demonstrated in various industries such as automotive, aerospace, electronics, shipbuilding, and railway, can be fully realized.

## Figures and Tables

**Table 1:** Chemical composition of 5182 aluminum alloy (wt%).

Si	Mg	Fe	Mn	Cu	Al
0.2~0.5	4.0~5.0	≤0.35	0.2~0.5	≤0.15	Bal.

**Table 2:** Mechanical property of 5182 aluminum alloy.

Material	Tensile Strength (MPa)	Yield Strength (MPa)	Elongation (%)	Elastic Modulus (GPa)	Brinell Hardness (HB)
AA5182	290~305	180~200	12~18	71	60~75

**Table 3:** Specific welding processing parameters utilized.

Welding Parameters	Value
Laser power (*p*), kW	3.0, 5.3
Welding speed (*v*), m/min	2.7, 5.4
Oscillation amplitude (*A*), mm	1.2, 1.6, 2.0
Oscillation frequency (*f*), Hz	120, 200, 250, 300
Tilt angle (*θ*), degree	20, 30, 40
Spot position	Upper, middle, lower parts of the upper plate’s side

**Table 4:** Types and standards of light-field distribution.

Shape	Type	Light-Field Distribution Standard
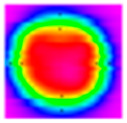	H	The *X*-axis red energy distribution area and the green energy distribution area are 74%, and the *Y*-axis is 70%.
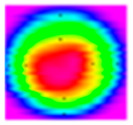	HH	The *X*-axis red energy distribution area and the green energy distribution area are 61%, and the *Y*-axis is 61%.
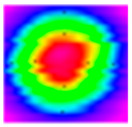	HHH	The *X*-axis red energy distribution area and the green energy distribution area are 50%, and the *Y*-axis is 58%.
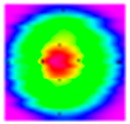	HHHH	The *X*-axis red energy distribution area and the green energy distribution area are 21.3%, and the *Y*-axis is 34.5%.

## Data Availability

The original contributions presented in this study are included in the article. Further inquiries can be directed to the corresponding authors.
